# Supplementary material for: Hydrophobic interactions of FG-nucleoporins are required for dilating nuclear membrane pores into selective transport channels after mitosis
Source: bioRxiv. 2025 Sep 8:2025.09.08.674396. Preprint. [Version 1] doi: 10.1101/2025.09.08.674396 (PMC12440036; doi:10.1101/2025.09.08.674396)
Supplement: Supplement 2 [file NIHPP2025.09.08.674396v1-supplement-2.pdf]

bioRxiv preprint doi: https://doi.org/10.1101/2025.09.08.674396; this version posted September 8, 2025. The copyright holder for this preprint (which was not certified by peer review) is the author/funder, who has granted bioRxiv a license to display the preprint in perpetuity. It is made available under aCC-BY 4.0 International license.

Supplementary Tables

Supplementary Table 1: Plasmids used in this study

Designation	Source or reference
pDiHcRed-NLS	Ellenberg Group
pmScarlet-Nup58	This work
pcDNA3.0_mitoLAMA-F98_IRES_mEGFP-RanT24N	This work

Supplementary Table 2: gRNA used in this study

bioRxiv preprint doi: https://doi.org/10.1101/2025.09.08.674396; this version posted September 8, 2025. The copyright holder for this preprint (which was not certified by peer review) is the author/funder, who has granted bioRxiv a license to display the preprint in perpetuity. It is made available under aCC-BY 4.0 International license.

Target Gene	Designation	Sequence
Nup62	Nup62C_gRNA_1-	TCGCtcaGTCAAAGGTGATCCGG

Supplementary Table 3: Antibodies used in this study

Antibodies	Dilution for immune-fluorescence	Dilution for Simple Western	Source or reference
Anti-Elys	1 to 50		Protein Atlas Antibodies, HPA031658
Anti-GFP		1 to 50	Abcam, ab290
Anti-Nup107		1 to 50	Proteintech, 19217-1-AP
Anti-Nup133	1 to 50		Abcam, ab155990
Anti-Nup153	1 to 500		Abcam, ab96462
Anti-Nup153		1 to 50	Abcam, ab245668
Anti-Nup155	1 to 100		Protein Atlas Antibodies, HPA037775
Anti-Nup188		1 to 50	Thermo Fisher, A302-322A
Anti-Nup214	1 to 100		Abcam, ab70497
Anti-Nup214		1 to 50	BETHYL, A300-716A
Anti-Nup358	1 to 100		Protein Atlas Antibodies, HPA018437
Anti-Nup54		1 to 25	Protein Atlas Antibodies, HPA035929
Anti-Nup58		1 to 25	Invitrogen, PA5-113478
Anti-Nup62		1 to 50	BD Biosciences, 610497
Anti-Nup88	1 to 50	1 to 25	BD Transduction Laboratories™, 611896
Anti-Nup98	1 to 50		Cell Signaling Technology, 2598S

bioRxiv preprint doi: https://doi.org/10.1101/2025.09.08.674396; this version posted September 8, 2025. The copyright holder for this preprint (which was not certified by peer review) is the author/funder, who has granted bioRxiv a license to display the preprint in perpetuity. It is made available under aCC-BY 4.0 International license.

Anti-Tpr	1 to 100		Protein Atlas Antibodies, HPA019661
Mab414	1 to 500	1 to 100	BioLegend, 902902
Anti-Rad21	1 to 500		Merck Millipore, 05-908
anti-Mouse-Abberior STAR 635P	1 to 250		Abberior, ST635P-1001-500UG
anti-Rabbit-Abberior STAR 580	1 to 250		Abberior, ST580-1002-500UG

Supplementary Table 4: Cell lines used in this study

**Designation**	**Source or reference**
HK WT	S. Narumiya (Kyoto University, Kyoto, Japan), RRID: CVCL_1922
HK Nup62-mEGFP-FKBP12^{F36V} #02	this work
HK Nup153-mEGFP-FKBP12^{F36V} #10-043	Brunner et al., 2025

bioRxiv preprint doi: https://doi.org/10.1101/2025.09.08.674396; this version posted September 8, 2025. The copyright holder for this preprint (which was not certified by peer review) is the author/funder, who has granted bioRxiv a license to display the preprint in perpetuity. It is made available under aCC-BY 4.0 International license.

Supplementary Figures

Zhang et al. Fig 1_Suppl 1

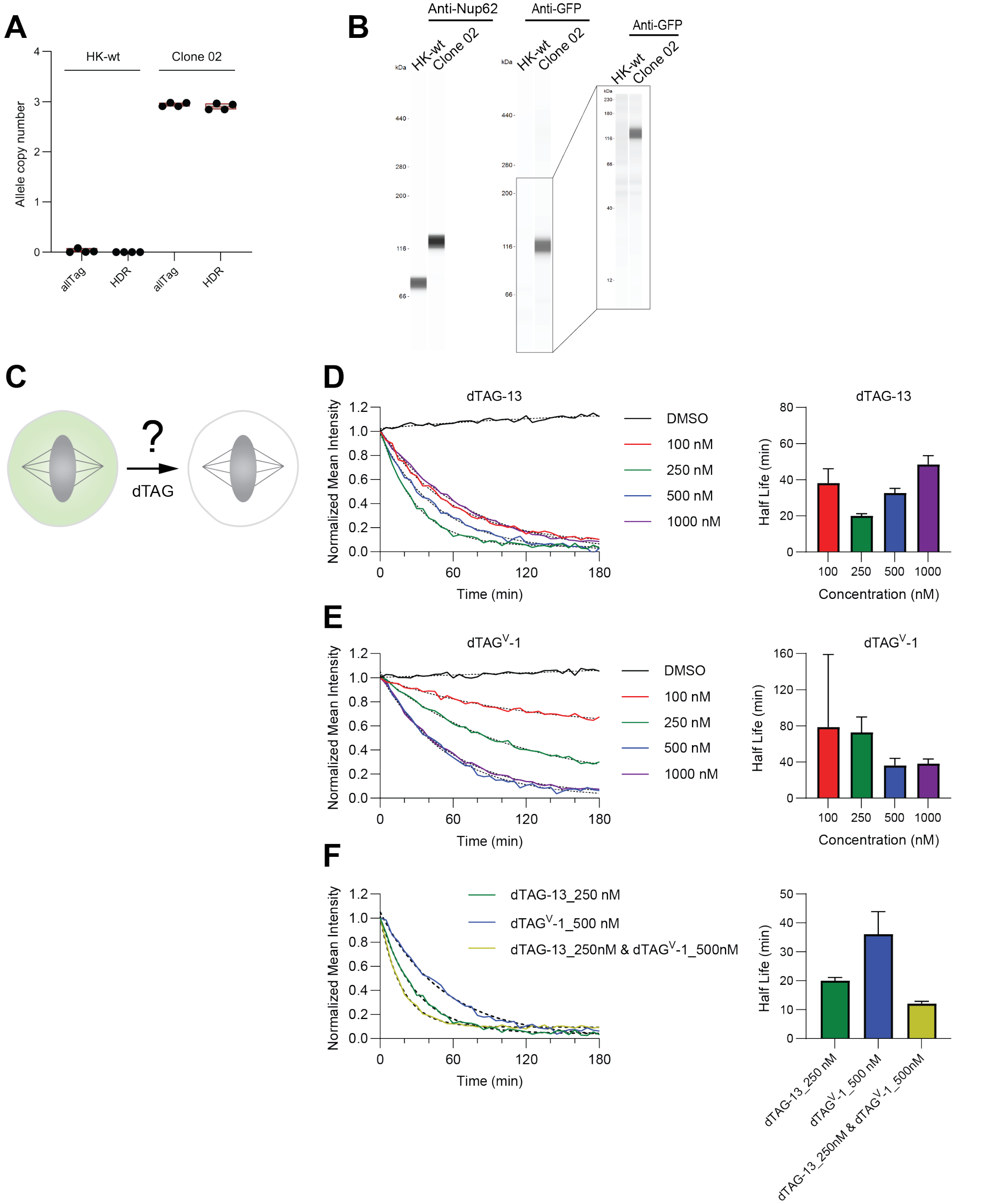

bioRxiv preprint doi: https://doi.org/10.1101/2025.09.08.674396; this version posted September 8, 2025. The copyright holder for this preprint (which was not certified by peer review) is the author/funder, who has granted bioRxiv a license to display the preprint in perpetuity. It is made available under aCC-BY 4.0 International license.

Figure 1_Supplementary 1 | Validation of the homozygous Nup62-mEGFP-FKBP12^{F36V} knock-in cell line and optimization of acute Nup62 depletion during mitotic arrest.

(A, B) Validation of the homozygous Nup62-mEGFP-FKBP12^{F36V} knock-in cell line (Clone 02). **(A)** Digital PCR analysis of homozygous integration of mEGFP-FKBP12^{F36V} at the Nup62 gene loci. Allele copy numbers of mEGFP-FKBP12^{F36V} integrations inserted into the HK genome (allTag) and at the endogenous Nup62 gene loci (HDR) were quantified from four replicates. Error bars indicate minimum and maximum values. **(B)** Simple Western analysis of total protein extracts from HK WT and the Nup62-mEGFP-FKBP12^{F36V} Clone 02 cells using antibodies against Nup62 and GFP. **(C)** Illustration of the assessment of Nup62-mEGFP-FKBP12^{F36V} degradation by dTAG compounds (dTAG-13 and/or dTAG^{V}-1) during mitotic arrest. **(D, E)** Kinetics analysis of Nup62-mEGFP-FKBP12^{F36V} degradation by titrating dTAG-13 and dTAG^{V}-1 concentrations. One-phase exponential decay models (dashed curves) were fitted to the data. Right panel: calculated half-life of degradation based on the fitted models. Sample sizes per condition: dTAG-13 (DMSO, n=13; 100 nM, n=7; 250 nM, n=9; 500 nM, n=12; 1000 nM, n=7) and dTAG^{V}-1 (DMSO, n=9; 100 nM, n=11; 250 nM, n=8; 500 nM, n=6; 1000 nM, n=8). **(F)** Combination of 250 nM dTAG-13 and 500 nM dTAG^{V}-1 (n=8) achieved the fastest degradation of Nup62-mEGFP-FKBP12^{F36V} among all tested conditions, as indicated by kinetic data and half-life calculated from fitted models (dashed curves).

bioRxiv preprint doi: https://doi.org/10.1101/2025.09.08.674396; this version posted September 8, 2025. The copyright holder for this preprint (which was not certified by peer review) is the author/funder, who has granted bioRxiv a license to display the preprint in perpetuity. It is made available under aCC-BY 4.0 International license.

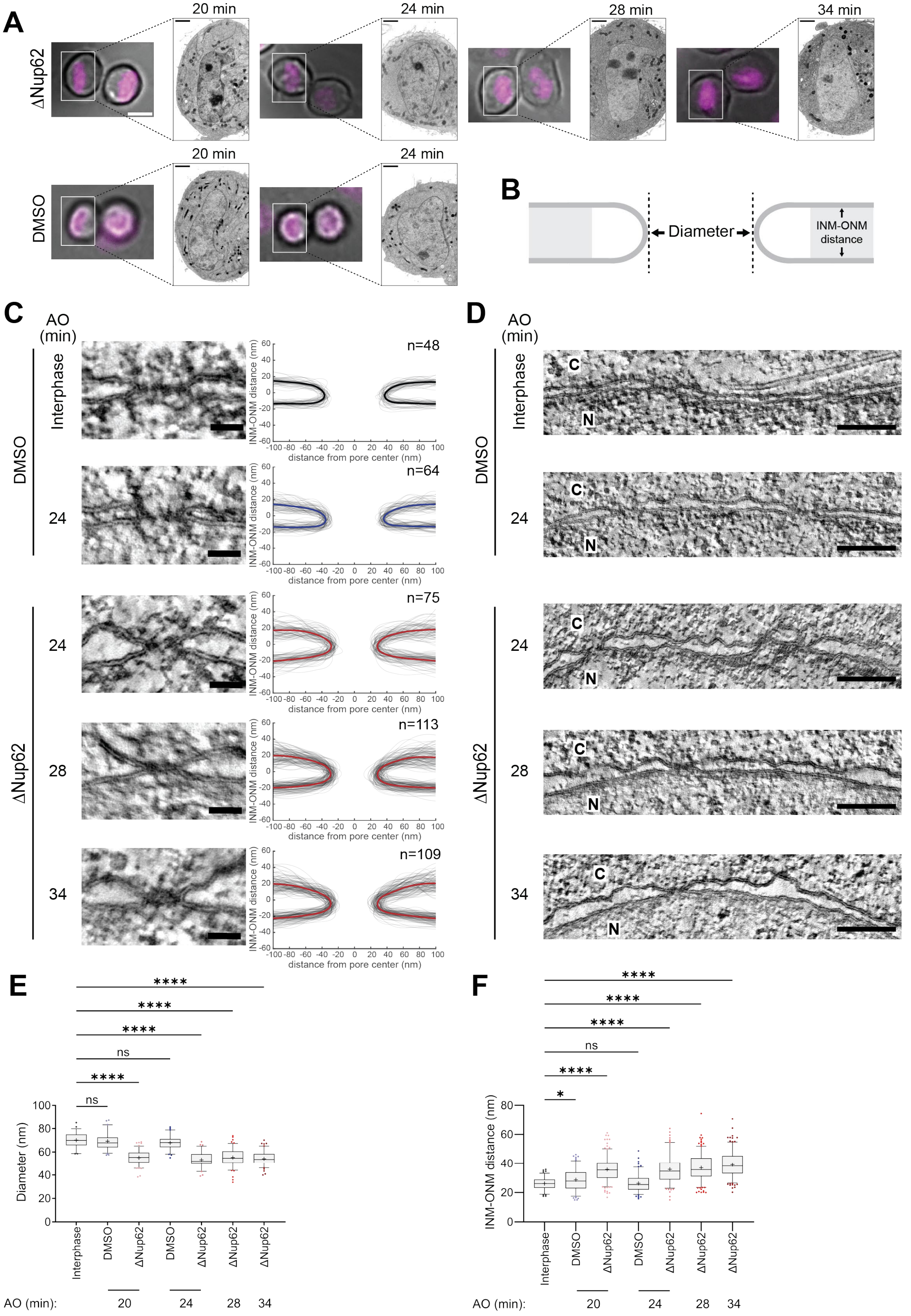

bioRxiv preprint doi: https://doi.org/10.1101/2025.09.08.674396; this version posted September 8, 2025. The copyright holder for this preprint (which was not certified by peer review) is the author/funder, who has granted bioRxiv a license to display the preprint in perpetuity. It is made available under aCC-BY 4.0 International license.

Figure 1_Supplementary 2 | Nup62 depletion during mitosis reduces assembled pore diameter and increases NE spacing, persisting at later stages after AO.

(A) Correlated live-cell and EM images of HK Nup62-mEGFP-FKBP^{F36V} cells analyzed by EM tomography. Cells treated with DMSO or dTAG (ΔNup62) were first imaged by light microscopy using live DNA dyes (shown in magenta) to track mitotic progression. The same cells were then subjected to high-pressure freezing, resin-embedding, serial sectioning, and observed in EM. Division time points after AO are indicated. Scale bars: light microscopy, 10 µm; EM, 2 µm. (**B)** Schematic illustrating how NPC diameter and INM–ONM distance were measured, indicated by bidirectional arrows. INM–ONM distance was calculated as the median of both sides of each measured pore within the indicated light gray areas (45-90 nm away from the pore tips). NPC diameter was measured as the distance between opposing membrane tips. **(C)** Left, tomographic slices showing cross-section views of nuclear pores from HK Nup62-mEGFP-FKBP^{F36V} cells treated with DMSO or dTAG (ΔNup62), at 24, 28, and 34 min after AO. NPCs from interphase cells treated with DMSO (> 180 min after AO) are shown for comparison. Right, membrane profiles of all measured pores at each time point are displayed, with mean profiles highlighted in bold. Scale bars, 50 nm. **(D)** Representative tomographic slices of the NE from the same samples as in (C). N, nucleus; C, cytoplasm. Scale bars, 200 nm. **(E, F)** Quantification of NPC diameter (E) and INM–ONM distance (F) from (C and D). Box-and-whisker plots show median, mean ("+"), 5–95 percentiles, and outliers (scatter). Sample sizes as indicated in (C) and Fig. 1B. Statistical significance applies to all panels in this figure: *, $P \leq 0.05$; **, $P \leq 0.01$; ***, $P \leq 0.001$; ****, $P \leq 0.0001$; ns, not significant.

bioRxiv preprint doi: https://doi.org/10.1101/2025.09.08.674396; this version posted September 8, 2025. The copyright holder for this preprint (which was not certified by peer review) is the author/funder, who has granted bioRxiv a license to display the preprint in perpetuity. It is made available under aCC-BY 4.0 International license.

Zhang et al. Fig 1_Suppl 3

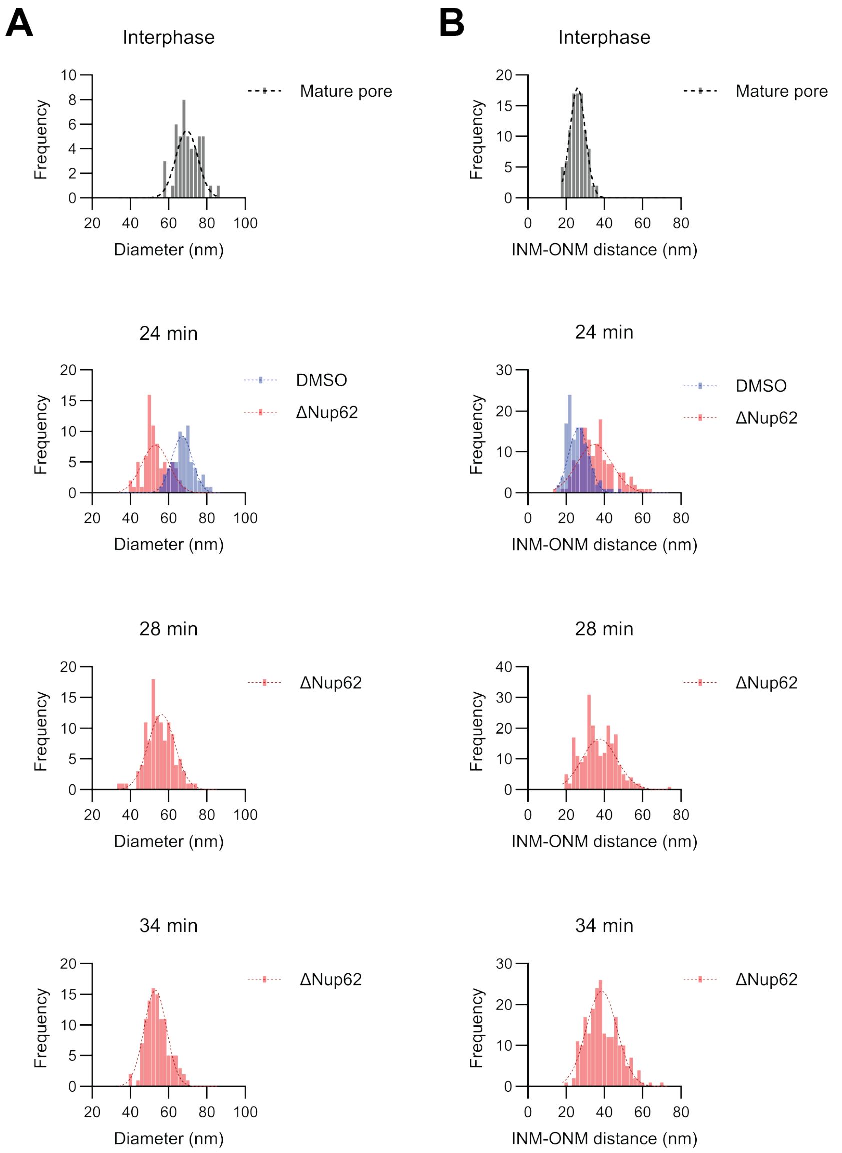

Figure 1_Supplementary 3 | Distribution of NPC diameter and INM-ONM distance measured in Fig. 1 Suppl. 2E and F.

(A, B) Histograms display the distribution of NPC diameter (A) and INM–ONM distance (B) at the indicated time points after AO, corresponding to Fig. 1 Suppl. 2E and F. Pores from interphase cells (> 180 min after AO) were analyzed for comparison (top panels). Gaussian distributions (dashed curves) were fitted to the histograms.

bioRxiv preprint doi: https://doi.org/10.1101/2025.09.08.674396; this version posted September 8, 2025. The copyright holder for this preprint (which was not certified by peer review) is the author/funder, who has granted bioRxiv a license to display the preprint in perpetuity. It is made available under aCC-BY 4.0 International license.

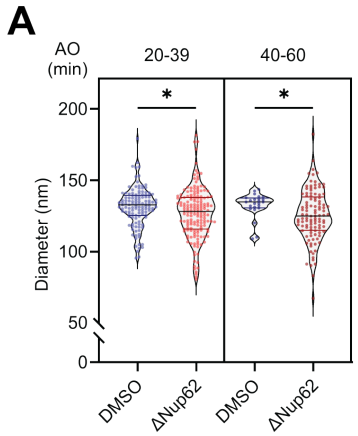

Figure 1_Supplementary 4 | Nup62 depletion during mitosis reduces nuclear outer ring diameter of assembled NPCs.

(A) Subgrouping of NPC diameter measurements according to the indicated time ranges after AO, corresponding to Fig. 1G. Median and quartiles are shown in the violin plot. Statistical significance applies to all panels in this figure: *, $P \leq 0.05$; **, $P \leq 0.01$; ***, $P \leq 0.001$; ****, $P \leq 0.0001$; ns, not significant.

bioRxiv preprint doi: https://doi.org/10.1101/2025.09.08.674396; this version posted September 8, 2025. The copyright holder for this preprint (which was not certified by peer review) is the author/funder, who has granted bioRxiv a license to display the preprint in perpetuity. It is made available under aCC-BY 4.0 International license.

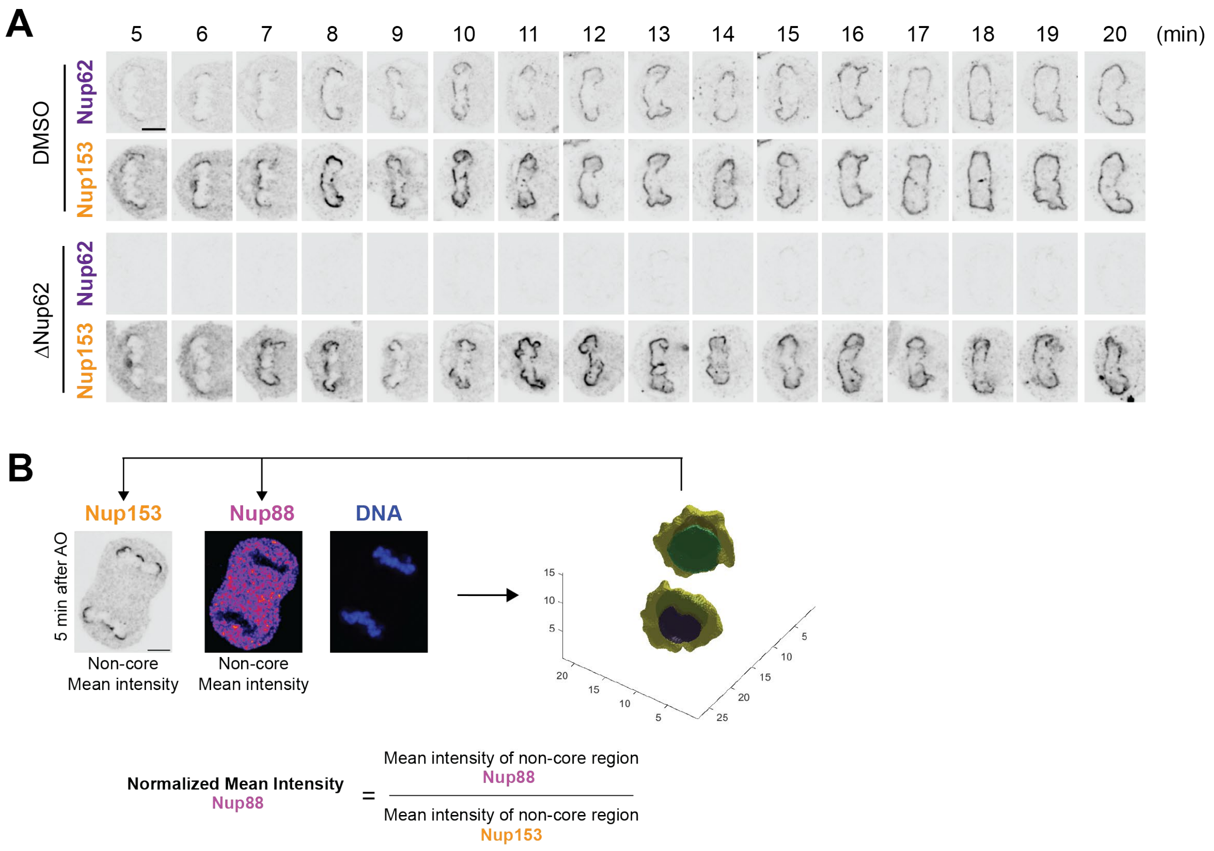

Figure 2_Supplementary 1 | Loss of Nup62 does not affect the recruitment of Nup153 during mitotic exit and illustration of quantitative analysis used to normalize Nups recruitment by Nup153.

(A) Representative images of HK Nup62-mEGFP-FKBP^{F36V} cells treated with DMSO or dTAG (ΔNup62), fixed at different time points (5-20 min) after AO, and stained with anti-Nup153 antibody. Nup62 signal is visualized via the mEGFP tagging. Scale bar, 5 µm. **(B)** Illustration of the normalized mean intensity calculation. A dividing cell at 5 min after AO, dually stained with anti-Nup153 and anti-Nup88 antibodies, is shown as an example. Scale bar, 5 µm. 3D segmented chromosomes based on the DNA channel and inferred non-core regions (yellow) are shown. Normalized mean intensity was calculated by dividing the measured mean intensity of Nup88 in non-core regions by the corresponding mean intensity of Nup153 measured in the same regions.

bioRxiv preprint doi: https://doi.org/10.1101/2025.09.08.674396; this version posted September 8, 2025. The copyright holder for this preprint (which was not certified by peer review) is the author/funder, who has granted bioRxiv a license to display the preprint in perpetuity. It is made available under aCC-BY 4.0 International license.

Zhang et al. Fig 2_Suppl 2

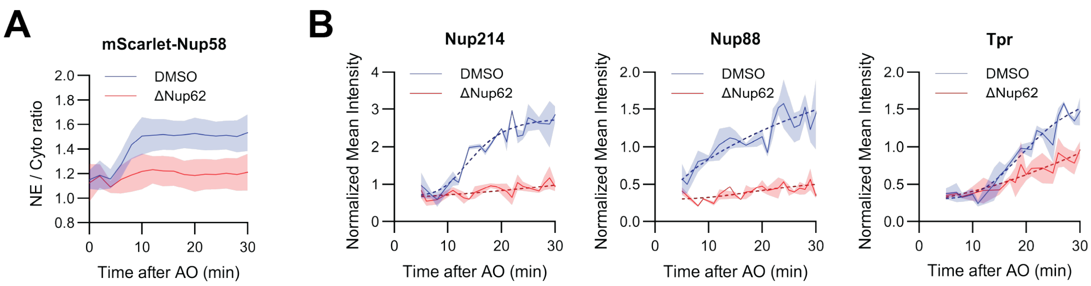

Figure 2_Supplementary 2 | Loss of Nup62 impairs the recruitment of its interacting partners and the nuclear basket component.

(A) Kinetic plot showing NE-to-cytoplasm mean intensity ratio of mScarlet-Nup58 in dividing HK Nup62-mEGFP-FKBP^{F36V} cells treated with DMSO or dTAG (ΔNup62) during mitotic exit (0-30 min after AO), corresponding to Fig. 2B. **(B)** Kinetic plots showing the normalized mean intensity of indicated Nups, measured from non-core regions of dividing HK Nup62-mEGFP-FKBP^{F36V} cells treated with DMSO or dTAG (ΔNup62), fixed at different time points (5-30 min) after AO, corresponding to Fig. 2C-E. Sigmoidal models (dashed curves) were fitted to the kinetic data.

bioRxiv preprint doi: https://doi.org/10.1101/2025.09.08.674396; this version posted September 8, 2025. The copyright holder for this preprint (which was not certified by peer review) is the author/funder, who has granted bioRxiv a license to display the preprint in perpetuity. It is made available under aCC-BY 4.0 International license.

Zhang et al. Fig 2_Suppl 3

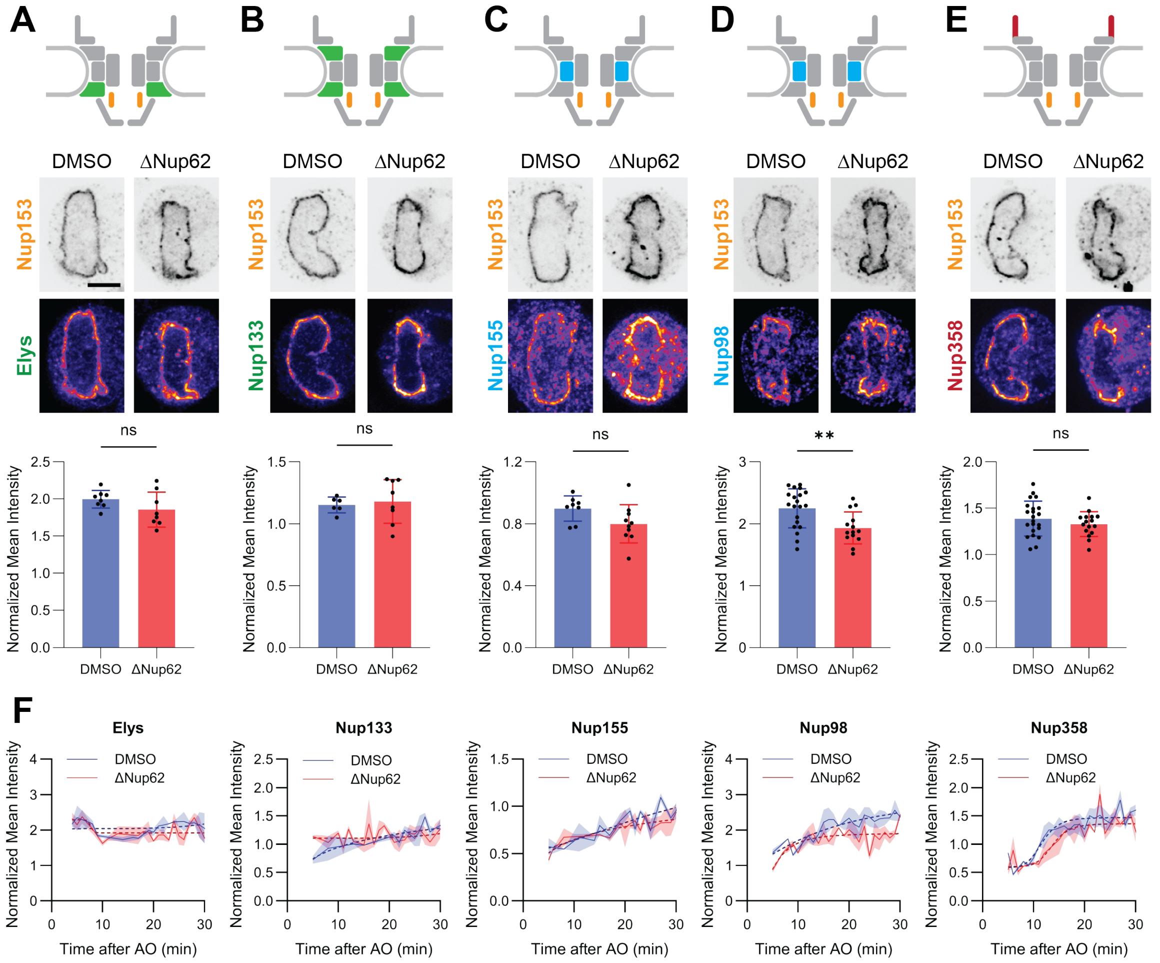

Figure 2_Supplementary 3 | Loss of Nup62 does not affect the recruitment of scaffold Nups and the cytoplasmic filament.

(A-E) Representative immunofluorescence images (middle panel) of HK Nup62-mEGFP-FKBP^{F36V} cells treated with DMSO or dTAG (ΔNup62) at 20 min after AO, dually stained with anti-Nup153 and anti-Elys (A), anti-Nup133 (B), anti-Nup155 (C), anti-Nup98 (D) or anti-Nup358 (E) antibodies. Upper panel: NPC schematic indicating the position of each labeled Nup within the complex. Lower panel: normalized mean intensity from non-core NE regions of dividing cells at 20 ± 1 min after AO. Scale bars, 5 µm. **(F)** Kinetic plots show the normalized mean intensity of indicated Nups shown in (C–G) calculated from non-core NE regions of cells fixed at different time points (5-30 min) after AO. Sigmoidal models (dashed curves) were fitted to the kinetic data. Statistical significance applies to all panels in this figure: *, P ≤ 0.05; **, P ≤ 0.01; ***, P ≤ 0.001; ****, P ≤ 0.0001; ns, not significant.

bioRxiv preprint doi: https://doi.org/10.1101/2025.09.08.674396; this version posted September 8, 2025. The copyright holder for this preprint (which was not certified by peer review) is the author/funder, who has granted bioRxiv a license to display the preprint in perpetuity. It is made available under aCC-BY 4.0 International license.

Zhang et al. Fig 2_Suppl 4

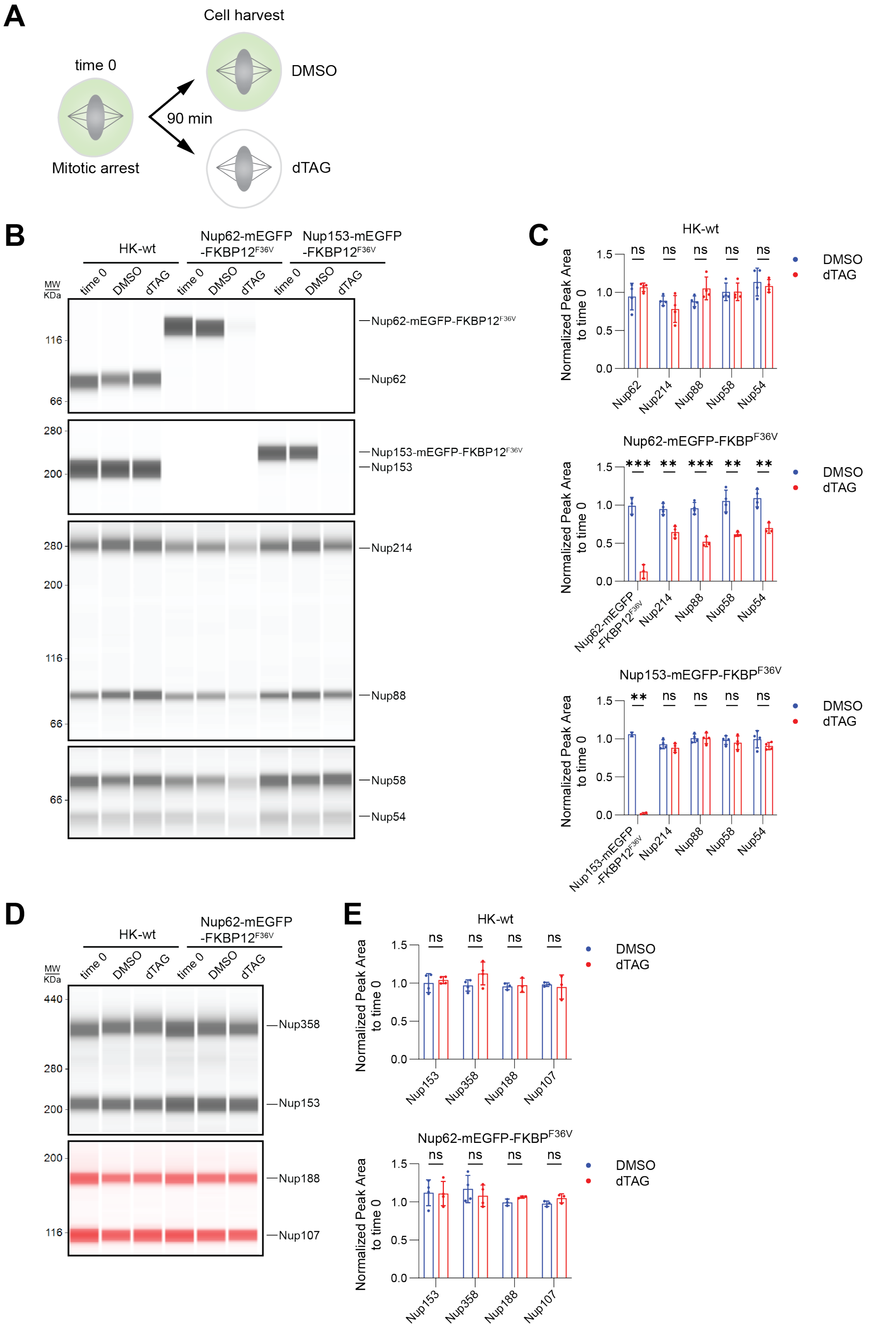

bioRxiv preprint doi: https://doi.org/10.1101/2025.09.08.674396; this version posted September 8, 2025. The copyright holder for this preprint (which was not certified by peer review) is the author/funder, who has granted bioRxiv a license to display the preprint in perpetuity. It is made available under aCC-BY 4.0 International license.

Figure 2_Supplementary 4 | Nup62 depletion leads to a decrease in the protein levels of its interacting Nups.

(A) Experimental setup for harvesting HK WT, HK Nup62-mEGFP-FKBP^{F36V} and HK Nup153-mEGFP-FKBP^{F36V} cells, arrested in prometaphase and treated with DMSO or dTAG (250 nM dTAG-13 and 500 nM dTAG^{V}-1) for 90 min. **(B, D)** Simple Western analysis of protein extracts from the indicated cell lines treated as described in (A). For each condition, 3 µl of total protein lysate (0.4 µg/µl) was loaded into the microplates. The Jess ProteinSimple capillary system was used to detect total loaded proteins via the protein normalization module and specific Nups using targeted antibodies. In (B), antibodies were used in the following order (top to bottom panels): anti-Nup62, anti-Nup153, anti-Nup214 & anti-Nup88, and anti-Nup58 & anti-Nup54. In (D), antibodies used were anti-Mab414 (detecting Nup358 and Nup153) and anti-Nup188 & Nup107 (detected with secondary NIR antibodies). **(C, E)** Quantification of normalized protein levels of Nups detected in (B and D). Protein levels were first normalized to total protein content detected by the Jess ProteinSimple protein normalization module, and then further normalized to time 0 to assess relative changes. Statistical significance applies to all panels in this figure: *, $P \leq 0.05$; **, $P \leq 0.01$; ***, $P \leq 0.001$; ****, $P \leq 0.0001$; ns, not significant.

bioRxiv preprint doi: https://doi.org/10.1101/2025.09.08.674396; this version posted September 8, 2025. The copyright holder for this preprint (which was not certified by peer review) is the author/funder, who has granted bioRxiv a license to display the preprint in perpetuity. It is made available under aCC-BY 4.0 International license.

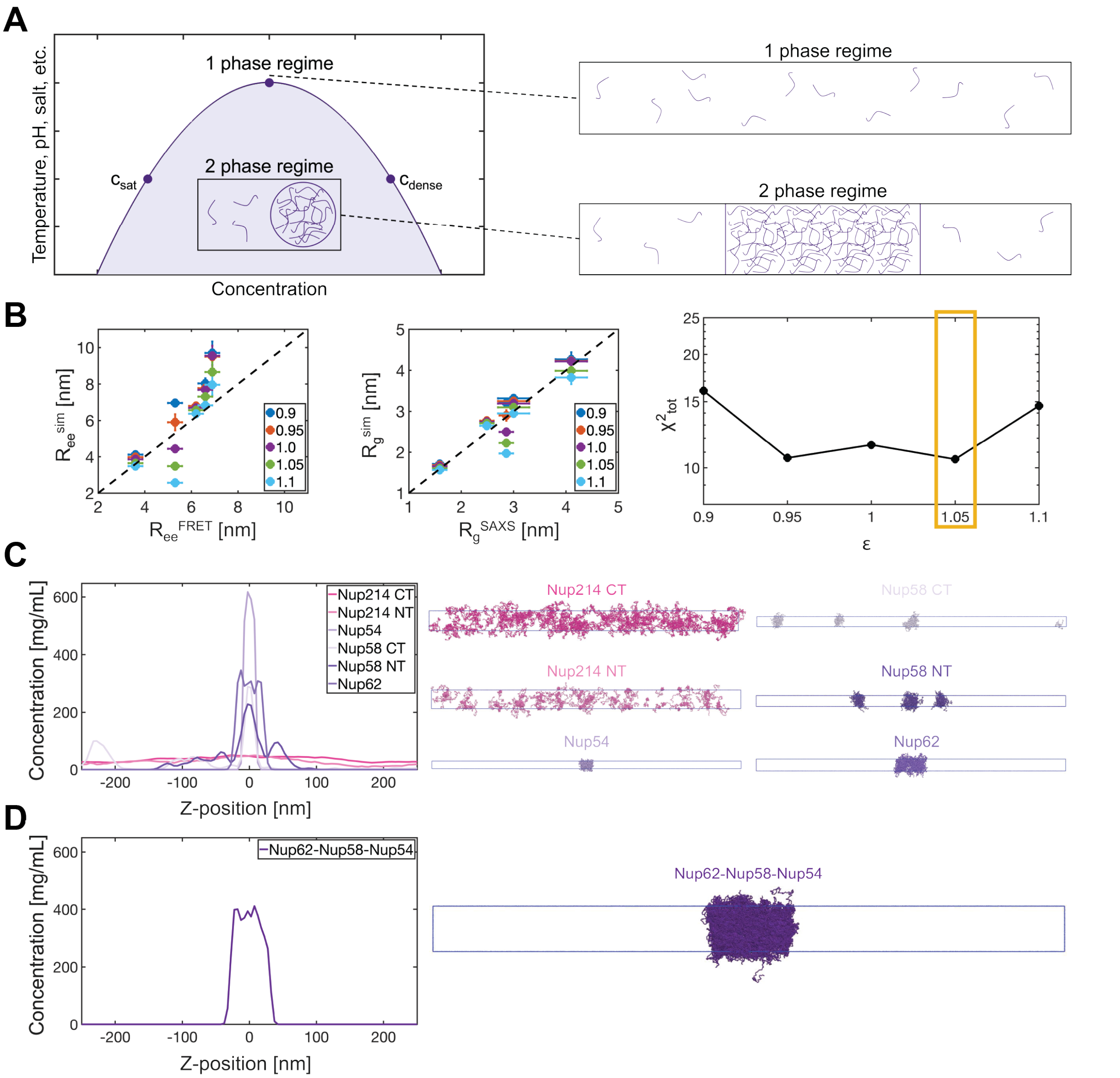

Figure 3_Supplementary 1 | Simulation of condensate formation by Nup62-58-54 and Nup214-88-62 subcomplexes.

(A) Illustration of a protein phase diagram. **(B)** Training MOFF to accurately model FG-Nups. Comparison between versions of MOFF with different ε to match experimental R_{ee} (left) or R_g (middle). Error bars in the X-axis represent standard deviations (SAXS) or the estimated lower bound of precision (FRET), while error bars in the Y-axis represent the standard deviation over five simulations with different random seeds. χ^2 as a function of ε is also shown (right), with the optimal ε value used in modeling boxed. **(C)** Slab density profiles as a function of box length for IDRs of Nup214, Nup54, Nup58, and Nup62 (left), with images of

bioRxiv preprint doi: https://doi.org/10.1101/2025.09.08.674396; this version posted September 8, 2025. The copyright holder for this preprint (which was not certified by peer review) is the author/funder, who has granted bioRxiv a license to display the preprint in perpetuity. It is made available under aCC-BY 4.0 International license.

the final frame from each simulation (right). **(D)** Slab density profile as a function of box length for the full length Nup62-Nup58-Nup54 subcomplex (left), with an image of the final frame from the simulation (right).

bioRxiv preprint doi: https://doi.org/10.1101/2025.09.08.674396; this version posted September 8, 2025. The copyright holder for this preprint (which was not certified by peer review) is the author/funder, who has granted bioRxiv a license to display the preprint in perpetuity. It is made available under aCC-BY 4.0 International license.

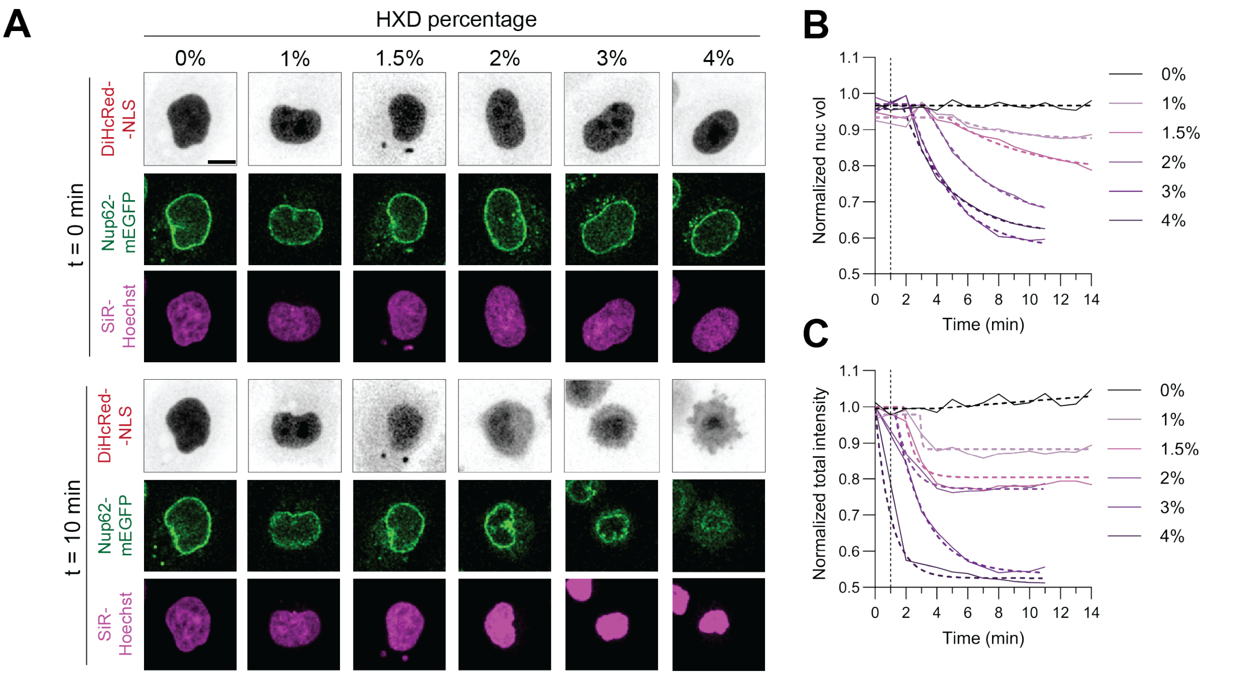

Figure 3_Supplementary 2 | Acute 1,6-hexanediol (HXD) treatment affects nuclear volume and nuclear import in interphase.

(A) HXD titration for acute disruption of FG-Nups condensate formation, assessed using the nuclear import reporter DiHcRed-NLS. HK Nup62-mEGFP-FKBP^{F36V} cells expressing DiHcRed-NLS and stained with SiR-Hoechst were imaged just before HXD addition (t = 0 min, upper panel) and after 10 min of treatment with the indicated HXD concentrations (lower panel). Scale bar, 10 μm. **(B, C)** Kinetic plots of nuclear volume (B) and total intensity of DiHcRed-NLS within the nucleus (C), normalized to t = 0, are shown over time. HXD was added at t = 1 min, as indicated by the dashed lines. Sample sizes per condition: 0%, n=14; 1%, n=14; 1.5%, n=10; 2%, n=12; 3%, n=11; 4%, n=12. Plateau followed by one-phase decay models (dashed curves) were fitted to the kinetic data.

bioRxiv preprint doi: https://doi.org/10.1101/2025.09.08.674396; this version posted September 8, 2025. The copyright holder for this preprint (which was not certified by peer review) is the author/funder, who has granted bioRxiv a license to display the preprint in perpetuity. It is made available under aCC-BY 4.0 International license.

Zhang et al. Fig 3_Suppl 3

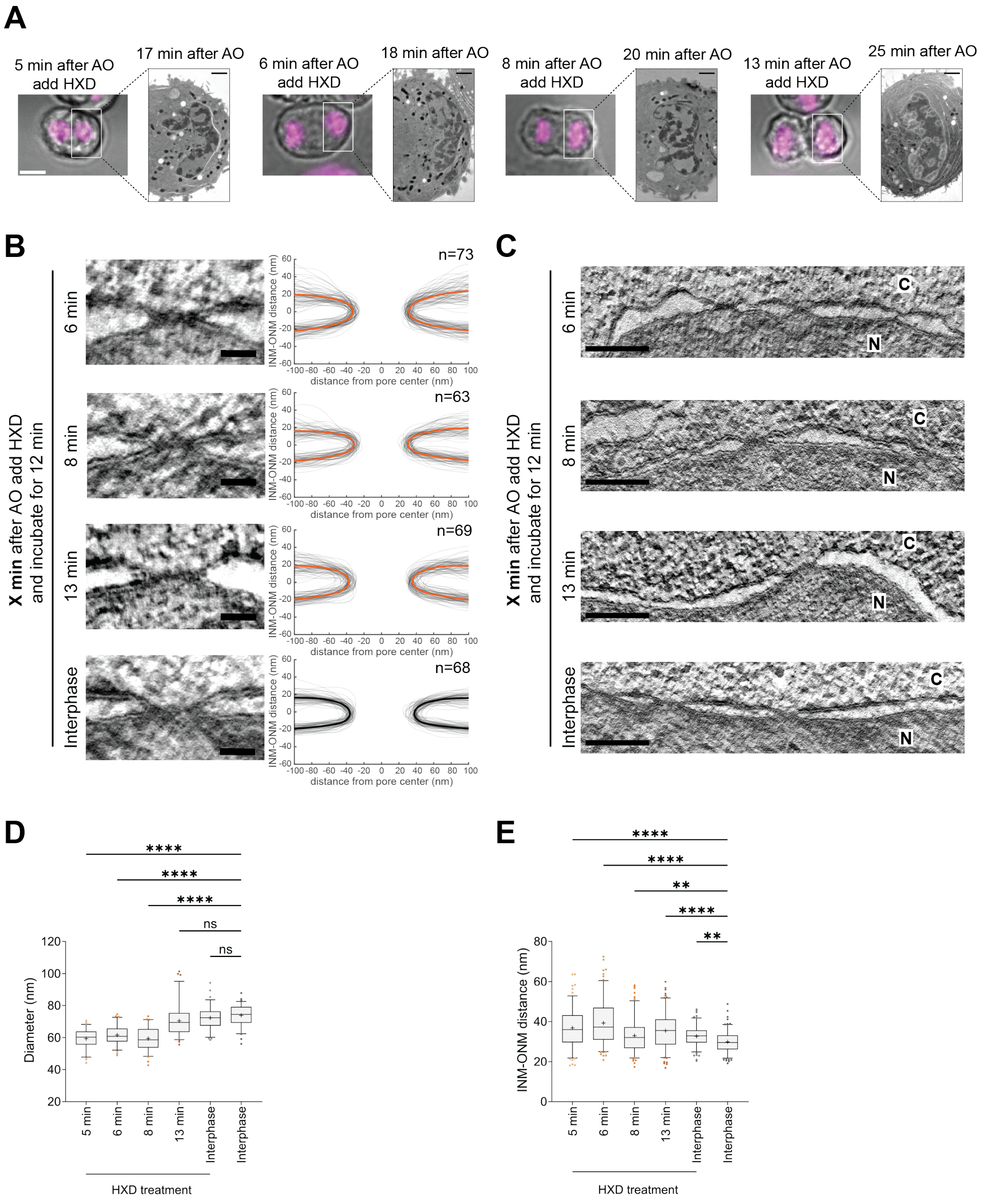

bioRxiv preprint doi: https://doi.org/10.1101/2025.09.08.674396; this version posted September 8, 2025. The copyright holder for this preprint (which was not certified by peer review) is the author/funder, who has granted bioRxiv a license to display the preprint in perpetuity. It is made available under aCC-BY 4.0 International license.

Figure 3_Supplementary 3 | Acute disruption of condensate formation before pore dilation during NPC assembly reduces NPC diameter and increases NE spacing.

(A) Correlated live-cell and EM images of HK WT cells analyzed by EM tomography. Cells were first imaged by light microscopy using live DNA dyes (shown in magenta) to track mitotic progression. Subsequently, 1.5% of HXD was added to cells at the indicated time points after AO and treated for 12 min. The same cells were then subjected to high-pressure freezing, resin-embedding, and serial sectioning, and observed in EM. Scale bars: light microscopy, 10 μm; EM, 2 μm. (**B)** Tomographic slices showing cross-section views of nuclear pores in HK WT cells treated with HXD at 6, 8, and 13 min after AO and in interphase. Membrane profiles of all measured pores are displayed, with mean profiles highlighted in bold. Scale bars, 50 nm. (**C)** Representative tomographic slices of the NE from the same samples as in (B). N, nucleus; C, cytoplasm. Scale bars, 200 nm. **(D, E)** Quantification of NPC diameter (D) and INM–ONM distance (E) from (C and D). Box-and-whisker plots show median, mean ("+"), 5–95 percentiles, and outliers (scatter). Sample sizes as indicated in (B) and Fig. 3E. Statistical significance applies to all panels in this figure: *, $P \leq 0.05$; **, $P \leq 0.01$; ***, $P \leq 0.001$; ****, $P \leq 0.0001$; ns, not significant.

bioRxiv preprint doi: https://doi.org/10.1101/2025.09.08.674396; this version posted September 8, 2025. The copyright holder for this preprint (which was not certified by peer review) is the author/funder, who has granted bioRxiv a license to display the preprint in perpetuity. It is made available under aCC-BY 4.0 International license.

Zhang et al. Fig 3_Suppl 4

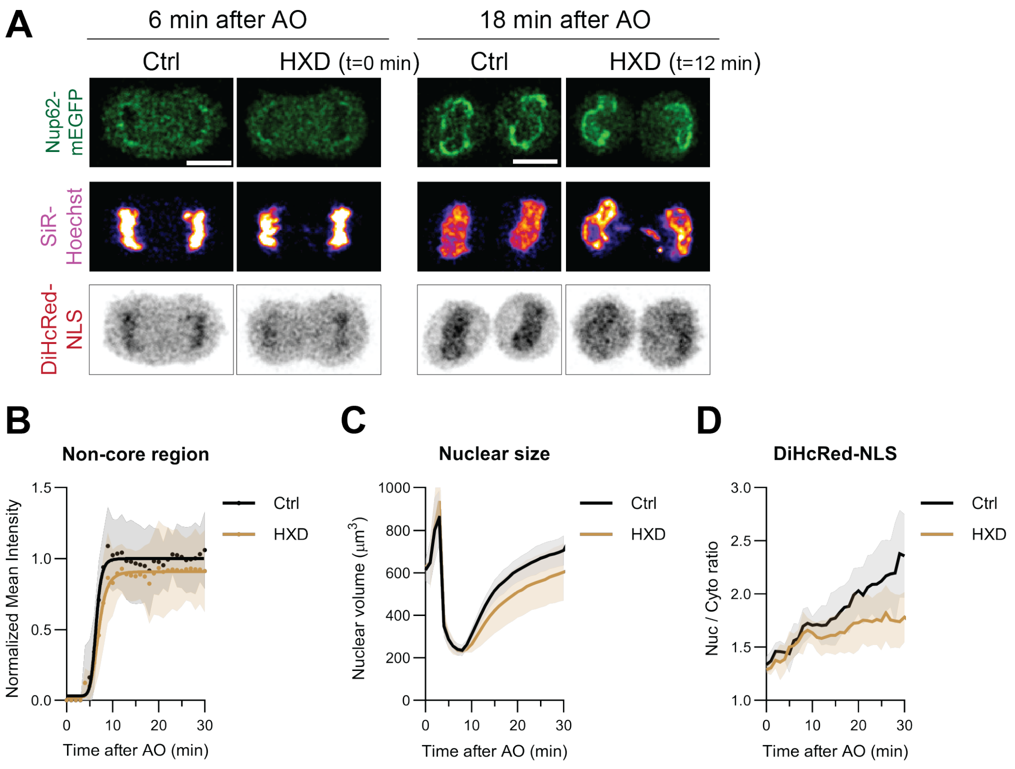

Figure 3_Supplementary 4 | Acute 1,6-hexanediol (HXD) treatment affects nuclear volume and nuclear import during mitotic exit.

(A) Representative live-cell images of HK Nup62-mEGFP-FKBP^{F36V} expressing DiHcRed-NLS at 6 min after AO, acutely treated with 1.5% of HXD for 12 min. Untreated cells served as controls. Scale bars, 10 µm. **(B)** Assembly kinetics of Nup62-mEGFP-FKBP^{F36V} in the non-core NE regions under control and HXD-treated conditions, from the same samples as in (A). Sigmoidal models (solid curves) were fitted to the kinetic data. Intensities were normalized to the plateau level of Nup62-mEGFP in the control condition. Data points represent the average normalized mean intensity, and error bands indicate the standard deviation. Sample sizes: Ctrl, n=16; HXD, n=12. **(C, D)** Kinetics of the average nuclear volume (C) and the nucleus-to-cytoplasm mean intensity ratio of DiHcRed-NLS (D) during mitotic exit, from the same samples as in (A). Error bands represent the standard deviation. Sample sizes: (C) Ctrl (n=16), HXD (n=12); (D) Ctrl (n=5), HXD (n=7).

bioRxiv preprint doi: https://doi.org/10.1101/2025.09.08.674396; this version posted September 8, 2025. The copyright holder for this preprint (which was not certified by peer review) is the author/funder, who has granted bioRxiv a license to display the preprint in perpetuity. It is made available under aCC-BY 4.0 International license.

Zhang et al. Fig 4_Suppl 1

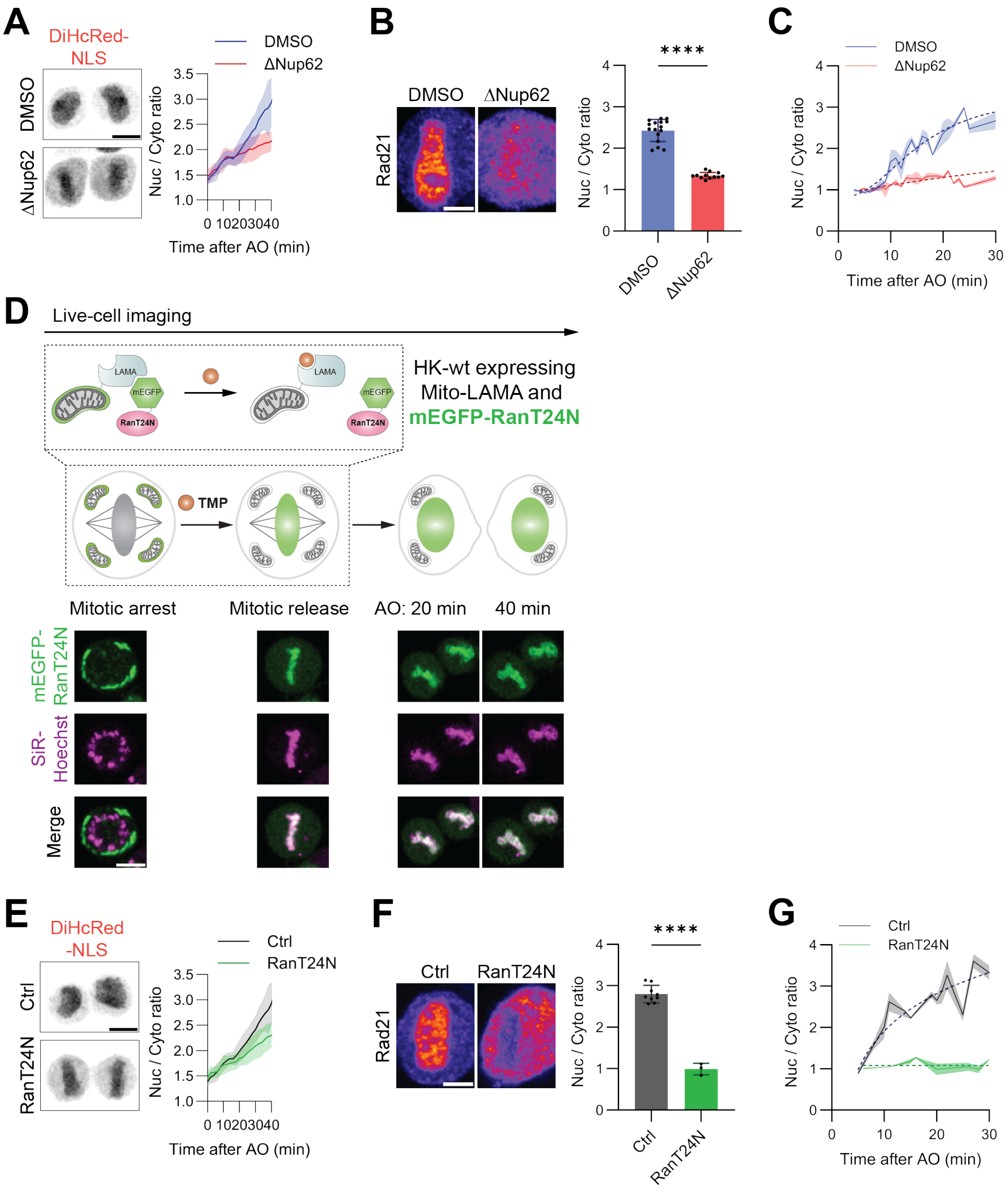

1378 **Figure 4_Supplementary 1 | Nup62 depletion and RanT24N activation during mitotic exit**
1379 **impair nuclear import.**

1380 **(A)** Representative live-cell images of HK Nup62-mEGFP-FKBP^{F36V} treated with DMSO or dTAG
1381 (ΔNup62), shown at 40 min after AO. Left, maximum projection of cells expressing DiHcRed-NLS. Scale
1382 bar, 10 µm. Right, nucleus-to-cytoplasm mean intensity ratio of DiHcRed-NLS measured in single cells
1383 during mitotic exit under the indicated conditions. Error bands represent the standard deviation. Sample

bioRxiv preprint doi: https://doi.org/10.1101/2025.09.08.674396; this version posted September 8, 2025. The copyright holder for this preprint (which was not certified by peer review) is the author/funder, who has granted bioRxiv a license to display the preprint in perpetuity. It is made available under aCC-BY 4.0 International license.

sizes: DMSO, n=10; ΔNup62, n=12. **(B)** Representative correlative immunofluorescence images of HK Nup62-mEGFP-FKBP^{F36V} cells treated with DMSO or dTAG (ΔNup62), stained with anti-Rad21 at 20 min after AO. Scale bar, 5 µm. Right, nucleus-to-cytoplasm mean intensity ratio of Rad21 in dividing cells at 20 ± 1 min after AO. **(C)** Kinetic plot showing the nucleus-to-cytoplasm mean intensity ratio of Rad21 shown in (B), fixed at different time points (5-30 min) after AO. Sigmoidal models (dashed curves) were fitted to the kinetic data. (**D)** Schematic of the experimental workflow for acute import inhibition via transient release of mEGFP-RanT24N into the nucleus from mitochondria during mitotic exit using the mito-LAMA system. Release was triggered by adding 50 µM of TMP (illustrated in the upper panel). An example of a cell co-expressing mito-LAMA and mEGFP-RanT24N undergoing mitosis, after the release of mEGFP-RanT24N into the nucleus, corresponding to Fig. 4B. Scale bar, 10 µm. **(E)** Representative live-cell images of HK WT cells under Ctrl or RanT24N conditions, shown at 40 min after AO. Left, maximum projection of cells expressing DiHcRed-NLS; scale bar, 10 µm. Right, nucleus-to-cytoplasm mean intensity ratio of DiHcRed-NLS measured in single cells during mitotic exit under the indicated conditions. Error bands represent the standard deviation. Sample sizes: Ctrl, n=14; RanT24N, n=17. **(F)** Representative correlative immunofluorescence images of HK WT cells under Ctrl or RanT24N conditions, stained with anti-Rad21 at 20 min after AO. Scale bar, 5 µm. Right, nucleus-to-cytoplasm mean intensity ratio of Rad21 in dividing cells at 20 ± 1 min after AO. **(G)** Kinetic plot showing the nucleus-to-cytoplasm mean intensity ratio of Rad21 shown in (F), fixed at different time points (5-30 min) after AO. Sigmoidal models (dashed curves) were fitted to the kinetic data.

bioRxiv preprint doi: https://doi.org/10.1101/2025.09.08.674396; this version posted September 8, 2025. The copyright holder for this preprint (which was not certified by peer review) is the author/funder, who has granted bioRxiv a license to display the preprint in perpetuity. It is made available under aCC-BY 4.0 International license.

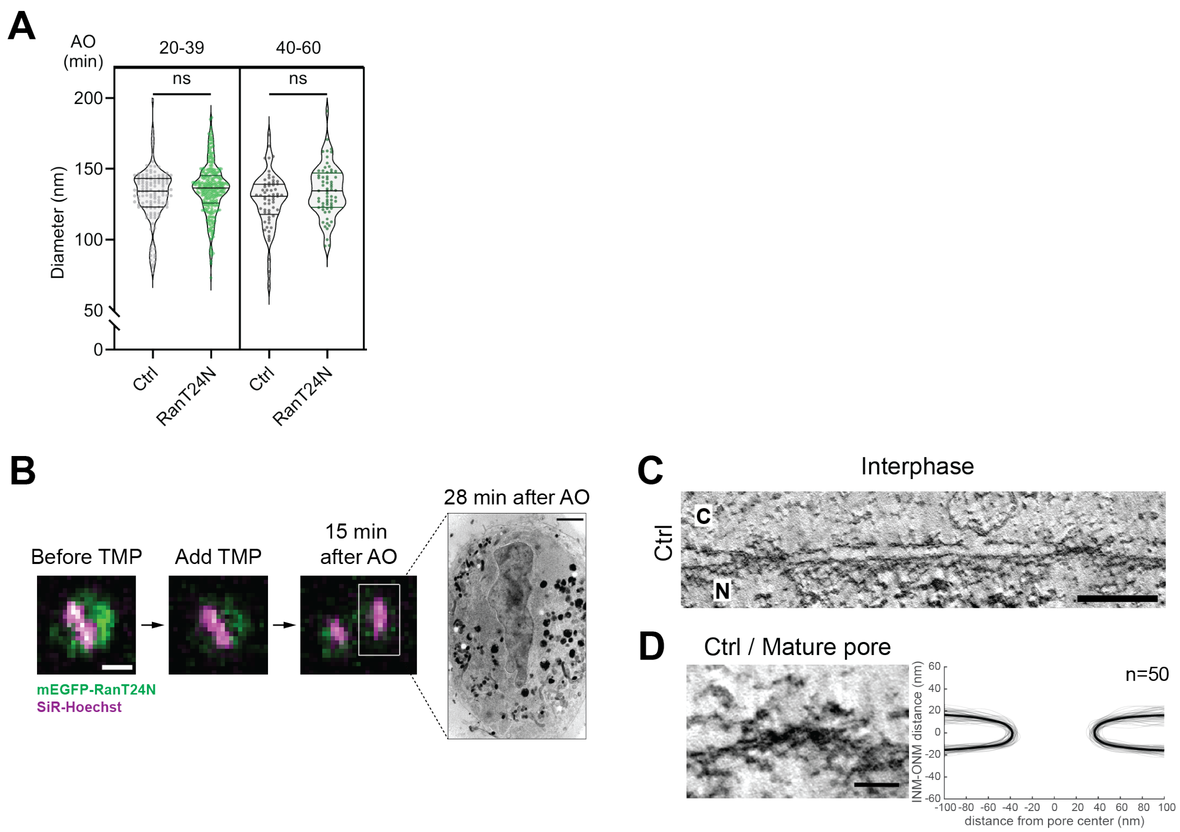

Figure 4_Supplementary 2 | RanT24N activation during mitotic exit does not affect NPC diameter.

(A) Subgrouping of pore diameter measurements according to the indicated division time ranges after AO, corresponding to Fig. 4E. Median and quartiles are shown in the violin plot. **(B)** Correlative live-cell and EM images of HK WT cells under RanT24N condition, analyzed by EM tomography. Cells were first imaged by light microscopy using live DNA dyes (shown in magenta) to track mitotic progression. Subsequently, 50 µM of TMP was then added to induce nuclear import of mEGFP-RanT24N during mitosis. The same cells were then subjected to high-pressure freezing, resin-embedding, serial sectioning, and observed in EM. Division time after AO is indicated. Scale bars: light microscopy, 10 µm; EM, 2 µm. **(C)** Representative tomographic slice of the NE in interphase HK WT cells under Ctrl condition. N, nucleus; C, cytoplasm. Scale bar, 200 nm. (**D)** Tomographic slices showing cross-section views of nuclear pores from the same samples as in (C). Membrane profiles of all measured pores are displayed, with mean profiles highlighted in bold. Scale bar, 50 nm. Statistical significance applies to all panels in this figure: *, $P \leq 0.05$; **, $P \leq 0.01$; ***, $P \leq 0.001$; ****, $P \leq 0.0001$; ns, not significant.
